# ReflACTION framework: A proposed model for implementation of clinical pharmacy services

**DOI:** 10.1016/j.rcsop.2024.100534

**Published:** 2024-10-22

**Authors:** Kérilin Stancine Santos Rocha, Sabrina Cerqueira-Santos, Genival Araújo dos Santos-Júnior, Lincoln Marques Cavalcante-Santos, Fernando de Castro Araújo-Neto, Fernanda Oliveira Prado, Giselle de Carvalho Brito, Divaldo Pereira de Lyra-Jr.

**Affiliations:** a Health Sciences Graduate Program. Laboratory of Teaching and Research in Social Pharmacy (LEPFS), Department of Pharmacy, Federal University of Sergipe, São Cristóvão, Sergipe, Brazil; b Graduate Program in Pharmaceutical Sciences. Laboratory of Teaching and Research in Social Pharmacy (LEPFS), Department of Pharmacy, Federal University of Sergipe, São Cristóvão, Sergipe, Brazil; c Research Group on Implementation and Integration of Clinical Pharmacy Services in Brazilian Health System (SUS), Department of Pharmacy and Nutrition, Federal University of Espírito Santo, Alegre, Espírito Santo, Brazil; d Research Center in Pharmaceutical Services and Clinical Pharmacy (CPAFF), Faculty of Pharmaceutical Sciences of Ribeirão Preto, University of São Paulo, Ribeirão Preto, São Paulo, Brazil; e Department of Health, State University of Feira de Santana, Feira de Santana, Bahia, Brazil; f Laboratory of Studies in Pharmaceutical Care, Department of Pharmacy, Federal University of Sergipe, Lagarto, Sergipe, Brazil; g Laboratory of Teaching and Research in Social Pharmacy (LEPFS), Department of Pharmacy, Federal University of Sergipe, São Cristóvão, Sergipe, Brazil; h Laboratory for Innovation in Health Care, Department of Pharmaceutical Sciences, Federal University of Espirito Santo, Vitória, Brazil; i Interdisciplinary Study Group on Pharmaceutical Care, Federal University of Juiz de Fora, Governador Valadares, Minas Gerais, Brazil

**Keywords:** Implementation science, Implementation framework, Quality improvement, Pharmaceutical services

## Abstract

The implementation of clinical pharmacy services (CPS) has grown worldwide. However, few studies have used models and/or frameworks to facilitate the implementation process, especially in a low and middle-income countries. In addition, there are limitations in the ways that implementation frameworks are used. Therefore, this discussion aimed to propose and describe an approach using the ReflACTION framework. ReflACTION emerged from several years of systematic observation and experience of the Laboratory of Teaching and Research in Social Pharmacy (LEPFS) in implementing CPS in different settings of Brazilian health system. These experiences led the research group to systematize the implementation of CPS based on three theorical references: Paulo Freire's theoretical references, the Maguerez Arc and the Apoteca framework. The ReflACTION framework proposes five steps that starts and ends in the setting, which are: observation of reality; gathering key-points; theorization; solution hypothesis; and application to reality. All steps were carried out considering the determinants of the implementation process. For the present study, we highlight the importance of the implementation team, the involvement of stakeholders as well as their dialogue and awareness. Thus, we describe the operationalization process for each step. The ReflACTION framework can be used to describe and guide the implementation process of CPS. We expect the proposed framework may add knowledge to implementation science and, ultimately, achieve desired patient outcomes.

## List of abbreviations

**Table:** 

CPS	Clinical Pharmacy Services

## 1 Introduction

The implementation of research into practice is a complex process with several factors that interact with each other and have an impact in the implementation processes and outcomes at different levels or domains.^1^^,^^2^ As a result, in the last decade, implementation science has observed a greater recognition in the need to establish theoretical references and strategies to facilitate this process.^1^^,^^3^^,^^4^ The use of theories, models and frameworks has emerged to achieve insights into the implementation process, and is essential to a successful implementation and long-term sustainability of the innovation.^4^^,^^5^

The field of implementation science has increased in Pharmacy practice in the past years.^6^ Among the models used to guide the implementation of an innovation within the Pharmacy field we can mention *The Promoting Action on Research Implementation in Health Services* (PARiHS) *framework,*^7^ the *Consolidated Framework for Implementation Research* (CFIR),^8^ the *Active Implementation Frameworks* (AIFs),^9^ and the *Framework for the Implementation of Services in Pharmacy* (FISpH).^10^ These frameworks are from other scientific disciplines, except the last one (FISpH) that was proposed to evaluate pharmaceutical services and their implementation programs.

Literature on describing the ways in which implementation frameworks are used and their theoretical and practical utility are often left unexamined.^11^^,^^12^ A recent scoping review on the implementation of Medication Review services in community pharmacies showed that only 9 (22 %) studies reported the use of a theoretical framework, model or tool in any stage of the research process to either plan the intervention, explain or contextualize the findings.^6^ In addition, none of the included studies was carried out in Latin America, Africa or Asia.^6^

Although these tools are essential in implementation science, it seems that their use is not known in the implementation of clinical pharmacy services (CPS), especially in low- and middle-income countries.^6^ Given this apparent gap, the development of a new framework that presents an implementation process that leaves no doubt on how frameworks are applied in research projects, and how they can support the implementation process may be useful. Therefore, this discussion aimed to describe an approach using the proposed model ReflACTION framework which may be helpful in the process of implementing CPS in different settings.

## 2 Contextualization

The present discussion proposed a tool to assist in the implementation of CPS, which we named ReflACTION framework. This framework is the result of 17 years of experience of the Laboratory of Teaching and Research in Social Pharmacy (LEPFS) in implementing CPS in different settings of the Brazilian health system and is based on consolidated theoretical references of the literature.

### 2.1 LEPFS experiences in implementing CPS

LEPFS belongs to the Department of Pharmacy of the Federal University of Sergipe, located in Northeast Brazil. The research group develops activities related to teaching, research and community actions, participation in policies drafting processes, and innovative practices in the country. Over 17 years of existence, the research group has conducted studies on the implementation of CPS at different healthcare levels and practice scenarios. Among the services implemented, it is worth mentioning the following: health screening, health education, drug dispensing, medication review, medication reconciliation, and comprehensive medication management. These services were implemented in the most diverse settings, such as primary care units, community pharmacies, long-term care institutions, specialized care, ambulatory care and hospitals.^13^, ^14^, ^15^, ^16^, ^17^, ^18^, ^19^, ^20^, 21., 22, 23, 24, 25, 26 Thus, all experiences led to the systematization of processes based on some theoretical references, presented below.

### 2.2 Theoretical references

The theoretical references that influenced the idea of the present framework were Paulo Freire's references ^27^, the Problematization with Maguerez Arc,^28^ and the Apoteca framework.^19^ Such references are presented below:

#### 2.2.1 Paulo Freire's theoretical references

Paulo Freire is one of the greatest minds in the history of world pedagogy, being a key reference in the education field. In the last four decades, he has also been referenced within the healthcare field, including Pharmacy, with an emphasis on observations between theory, practice, and fundamental values  related to patient empowerment and participation.^29^^,^^30^ His works present two concepts that influenced the perspective of the model presented in the present study: dialogue/dialogicity and awareness/critical awareness. Dialogicity has the dialogue as a central element. According to the author, subjects get to know and transform the world in collaboration through the dialogue. In turn, awareness refers to the process of building critical awareness. Paulo Freire states that the subject can create, choose, decide, commit, and transform reality and himself through awareness. Thus, the subjects' reflection and action on the world is of great importance, which require critical insertion to act on it.

#### 2.2.2 The Problematization with the Maguerez Arc

**Fig. 1:**
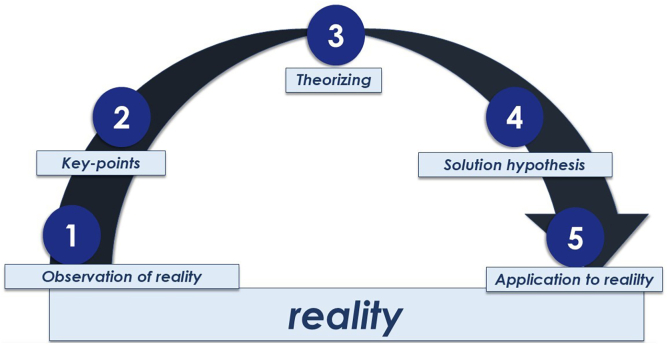
Maguerez Arc used by Berbel and Gamboa (2011).

The Problematization with the Maguerez Arc is a method focused on social transformation.^28^ This theoretical reference has the advantage of using an active work to exercise the detection of problems from reality, critically reflect on them and return to reality with potentially transformative interventions. This method allows the exercise of the chain *action-reflection-action*, taking social reality as the starting and the end point. In addition, it has been used both in teaching^31^^,^^32^ and research.^33^^,^^34^ The Problematization with Maguerez Arc has five steps (Fig. 1):

• Observation of reality: provides the identification of what needs to be investigated, resolved and improved so that reality may be transformed;
• Key-points: provides the definition of the problems observed in the previous step. It aims to raise essential points that should be studied and understood more deeply;
• Theorization: promotes the search for answers to the identified problems in the literature, so that theoretical references may assist in the elaboration of strategies to solve these problems;
• Solution hypotheses: promotes the elaboration of possible solutions for the identified problems based on the theoretical references of the previous step;
• Application to reality: provides the implementation of the defined strategies and interventions to promote changes in the studied reality.

#### 2.2.3 Apoteca Framework

**Fig. 2:**
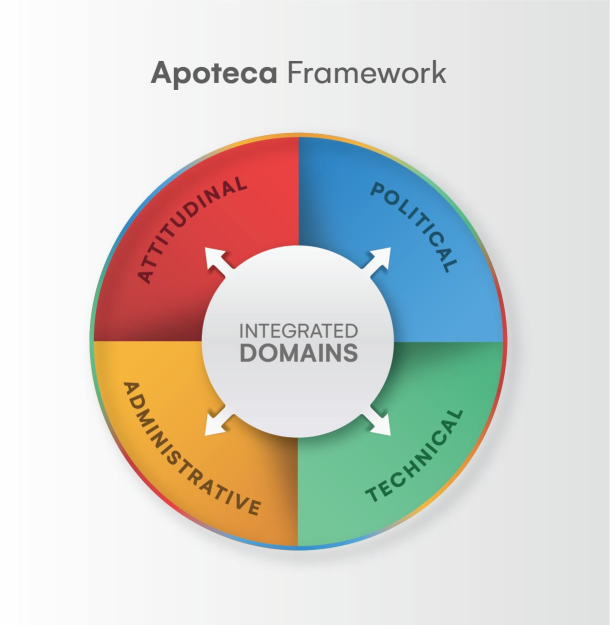
Apoteca Framework adapted.

Apoteca is a determinant implementation framework proposed by LEPFS.^21^^,^^35^ This framework was designed to assist in understanding the factors that influence the implementation of CPS, generating insights on strategies to consolidate these services. Four domains compose Apoteca: Attitudinal, POlitical, TEChnical and Administrative (Apoteca), defined as follows (Fig. 2):

• Administrative domain: related to administrative processes (organization and management) that are required to perform CPS.
• Attitudinal domain: related to behavior, action, or reaction, and motivated by a feeling or opinion towards a particular fact or person. In addition, internal and external motivation.
• Political domain: related to the relationships within a group or organization that allow individuals or groups to influence others (support).
• Technical domain: related to the CPS's own characteristics as well as the skills and knowledge needed to perform them.

### 2.3 ReflACTION framework development

The ReflACTION framework emerged from a series of discussions between the researchers of LEPFS who had been through the experiences previously described, and used the mentioned theoretical references separately ^21,34,35^. According to Varpio et al. (2020), theoretical frameworks are structures that connect data and theories that are related to each other, whether to hypothesize or drive innovative processes in the field of knowledge.^36^

In this perspective, Onozato et al. (2020)^35^ used the Apoteca Framework in a systematic review to categorize the factors that influenced the implantation of CPS for hospitalized patients. In addition, Santos Júnior et al. (2021)^34^ used the Problematization with the Maguerez Arc to evaluate the CPS implementation in public healthcare units of a metropolis in Northeast Brazil. The study also used the Apoteca framework to plan the necessary interventions for the CPS implementation. From reflections and insights of the previous research, the authors of the present study realized the need to combine these two tools for contemplation of the entire implementation process.

The combination of the Problematization with the Maguerez Arc with the Apoteca framework was possible due to the complementary objectives of the implementation process. The Maguerez Arc has a prescriptive character, that is, aims to provide direction on the implementation process via a series of steps or procedures. The Apoteca framework, in turn, is a framework of determinants, that is, identifies the determinants (factors, barriers and facilitators) that influence the implementation processes and outcomes and may be used at all steps of the implementation process.

Therefore, the ReflACTION framework may guide the implementation process of an innovation through steps, starting from the practice scenario (i.e. setting), and from reflexive, dialogical and awareness-raising processes return to it through potentially transformative actions, considering the determinants of the implementation in all steps.

#### 2.3.1 Actors involved in the operationalization of the ReflACTION framework

By being a tool that provides dialogicity and awareness, the engagement of actors is essential for the entire implementation process. It is important to highlight that the selection of actors should be flexible to meet the particularities of the practice scenario and the CPS to be implemented. However, we recommend that the roles of each member should be defined before starting of the implementation process. All actors and their functions in the proposed tool are described below.^34^^,^^35^^,^^37^, ^38^, ^39^

• Stakeholders: persons or groups who may be directly/indirectly affected by or influence the implementation process. These include, but are not limited to, a person, patients, pharmacists, healthcare workers, health system managers, health policy-makers communities, and organizations.^35^^,^^40^^,^^41^
• Implementation team: working group responsible for conducting the implementation processes, ensuring the supportive environment and facilitating the implementation of innovations using implementation science frameworks / theoretical references. They can be both internal and external to the institution. They are responsible for conduct and execute all steps related to the implementation process, must be always in constant dialogue with stakeholders to raise their awareness process, using implementation science theoretical references.^38^^,^^39^
• Coordinator: member of the implementation team, may have a political character, with experience in implementing innovations, who will lead the process. The coordinator promotes articulation and engagement between the management team and other actors involved in the process, in addition to planning and monitoring activities.^17^
• Supporters: pharmacists who are specialists in clinical pharmacy, with experience in implementing innovations, who will provide technical and attitudinal support to the pharmacists involved with the implementation of an innovation. They are responsible for promote training, follow-up and monitoring of clinical services performed by pharmacists. They will make the implementation process easier, help the pharmacists in achieving goals, encourage and promote action, help them to change their attitudes, habits, skills, ways of thinking, and working to incorporate evidence into practice.^21^^,^^39^

## 3 Description of the ReflACTION framework

The proposed model is divided into five steps that starts and ends in the practice scenario (reality) to be assessed. Throughout the results section, each of these steps will have a suggestion on how they can be carried out. As this framework is intended to be flexible, the implementation process might be adapted according to the practice scenario and the defined objective. Other alternatives that are not mentioned in this work may be used, since the framework intention is not to cover all the alternatives, but to point out the possibilities. The steps are detailed below:

### 3.1 Observation of reality

This step aims to gather problems of the studied reality and the conditions necessary for starting the implementation process. Thus, this step provides the identification of what needs to be investigated, resolved and improved so that reality may be transformed. For CPS implementation, the implementation team must systematically observe and record what they notice about the practice scenario in which the implementation is about to happen. Such observation will allow the team to identify difficulties, deficiencies, discrepancies, facilitators, among others, and they will be all problematized later. Thus, the administrative, political, technical and attitudinal factors that may interfere in the implementation process should be identified. This will make it possible to observe which domains of the Apoteca framework may influence to a greater or lesser extent the CPS implementation in that reality.^19^^,^^21^^,^^34^^,^^35^

Quantitative and qualitative approaches might be used to achieve these results. Among the quantitative approaches, we can highlight the investigation of structure, process and outcome indicators.^42^^,^^43^ Among the qualitative methods, we can mention approaches such as ethnography^44^ or even when there is no specific approach, qualitative data collection may be used to allow a more comprehensive understanding of that reality, such as values, culture, feeling about changing, among others. At this point, it is important to define all indicators that will be used.^45^ During this process, the implementation team should be in constant dialogue with stakeholders, to raise their protagonist, so that the reality may be better understood from the point of view of these actors and, therefore, transformed.

### 3.2 Key-points

This step aims to elaborate the essential points that must to be studied on the problem. Thus, from the observation of reality, the implementation team in dialogue with stakeholders should deliberate on what was observed, on the possible causes of the existence of such problems and the identified facilitators. Based on this reflective analysis, they should elaborate essential points that should be studied about the problems, to understand it in greater detail.^18^^,^^39^ Moreover, they should find ways to interfere in the studied reality aiming to solve such problems or trigger steps in that direction. Therefore, at this stage, the implementation team must understand the factors that contribute and trigger difficulties, and those that generate opportunities and drive changes in that reality in favor of implementation.

The identified key points should be in line with the determinants proposed by the Apoteca framework, that is, the implementation team should ask itself about which administrative, political, technical and attitudinal factors can positively and negatively influence the implementation process.^21^^,^^35^^,^^37^ From this, the key points will be defined and can be expressed in the form of statements, questions or topics. For gathering the key points, creative techniques like brainstorming and brainwriting may be helpful alternatives to be used with the implementation team.

### 3.3 Theorization

This step aims the confrontation of the key points, defined in the previous step, with the best available scientific evidence. Therefore, for theorization, the implementation team will search in the literature for answers to the problems identified. In general, problems extracted from reality are complex, challenging and multidimensional, which will demand the study of these problems in different angles and aspects.^16^^,^^26^^,^^46^^,^^47^ Such assessment will facilitate the process of dealing with them according to their complexity and achieve quite a few solution hypotheses. To this end, the implementation team may search for evidence in implementation studies, as well as in models and frameworks described in databases (e.g. Pubmed and Embase), using appropriate key words.^18^^,^^20^^,^^34^^,^^37^^,^^39^^,^^48^^,^^49^ There is no ideal formula since the search will depend on the problems of each studied reality. Thus, it is possible that some answers will be outside the scope of pharmacy and permeate fields in administration, psychology, education, among others.

The implementation team should discuss the evidence found and think about it considering three aspects: the best available evidence, previous experiences of the implementation team, and the needs of reality and stakeholders. This step will allow the theoretical references to assist in the elaboration of strategies in the attitudinal, political, technical and administrative domains, which seek the transformation of reality. The information obtained should be treated, analyzed and assessed in terms of its contributions to solving the problem in search of positive outcomes. As in other steps, it is important that stakeholders are involved in this process, so that the strategies are feasible to be incorporated into the reality in question.^40^^,^^41^

### 3.4 Solution hypotheses

This step aims to provide possible solutions to the identified problems and to boost the implementation process. For that matter, the implementation team, in dialogue with the stakeholders, will elaborate the possible solutions based on the theoretical references raised in the previous step and the reflections made.^34^^,^^37^ At this point, there will be planning, organization and evaluation of the best strategies that will produce the change, due to the deep understanding that was obtained about the problem in the previous step. These solutions are methods or techniques used to enhance the implementation process and provide sustainability of the innovation. Typically, a single strategy is useful for dealing with different barriers; however, the use more than one strategy to deal with a single barrier is common. Moreover, it is important to highlight that these interventions should be structured and reproducible, as well as based on scientific evidence and on the implementation team previous experiences and the stakeholders' preferences.

Each member of the implementation team must be responsible for executing one or more solution hypotheses, so they can be allocated according to the Apoteca framework and facilitate the implementation process. Some consolidated tools from the literature may be used to assist this step such as the PDSA (P: plan, D: do, S: study, and A: act.) and the 5W3H (What, Why, Where, When, Who, How, How much, How many).^39^^,^^49^^,^^50^ Schemes of prioritizing strategies may be used in addition such as the Nominal Group Technique, Basic Matrix, among others. A previous study described some solution hypotheses for CPS implementation.^23^^,^^30^

### 3.5 Reality application

This step aims to apply the previously established strategies into reality. Thus, the application will be carried out by the implementation team at different moments of the process, according to the needs of each practice scenario and using as reference the general overview that the Apoteca framework provided.^21^^,^^35^^,^^51^ This step will help the implementation team and the stakeholders to execute the planned interventions and, consequently, seek the transformation of practices and optimization of processes.

### 3.6 Preliminary assessment – New observation of reality

**Fig. 3:**
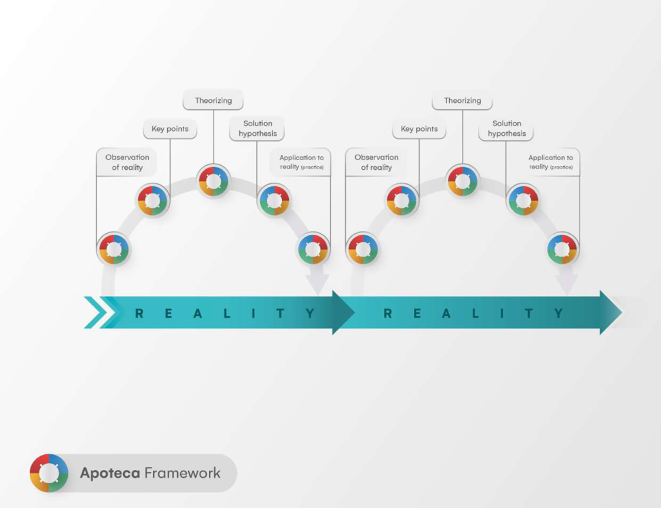
The ReflACTION Framework.

The ReflACTION Framework has a cyclical character, that is, at the end of the five steps, a new cycle can be started (Fig. 3). Therefore, it is recommended that all steps of the ReflACTION Framework be carried out again until the reality is positively modified, and the desired results are achieved. The ultimate goal is to accomplish the sustainability of innovations and, consequently, promote their continuity and long-term integration.

With the cyclical application of the framework, a new observation of reality will occur, in which new or old implementation factors can be identified, problematized and interventions proposed and applied again. Throughout this process, the defined indicators should be monitored to allow assessments, strategy design and reassessments. Thus, monitoring the results will facilitate assessing the impact of the implementation so far, and promote the process moves towards the sustainability of the service.

### 3.7 Framework usability

The proposed framework was used to implement different CPS in different scenarios of the Brazilian healthcare system. In 2019, The Brazilian Health Ministry used concepts from the framework to implement the comprehensive medication management service for patients undergoing treatment for Rheumatoid Arthritis. The project was developed in partnership with the Health Secretariats of the States of São Paulo, Pernambuco, Federal District and Minas Gerais together with the Ministry of Health. This model is currently being used to implement drug dispensing service in a community pharmacy chain in a city in Northeast Brazil and in a public pharmacy in a city in Southeastern Brazil.^34^^,^^37^^,^^38^^,^^52^, ^53^, ^54^

## 4 Discussion

The use of models and frameworks that provide a structured and theoretical approach to implementation processes and outcomes may lead to implementation success.^5^ While some developed countries are at a more advanced stage, such as assessing the challenges to ensure the sustainability of innovations, low and middle income countries still seem to face initial challenges inherent to the implementation process.^55^^,^^56^

Therefore, we believe that the model proposed in this study may be particularly useful for countries that face challenges like those in Brazil. This country has continental dimensions and deep economic, social and regional inequalities. The Brazilian Healthcare System is configured as one of the largest and most complex public healthcare systems in the world, which guarantees access to health in a comprehensive, universal and free manner for the entire population.^57^ In addition, the reality of the pharmaceutical profession in Brazil is challenging. Although the clinical performance of pharmacists has been stimulated in recent years, it remains heterogeneous in the country, being incipient in many practice scenarios.^58^, ^59^, ^60^

Choosing Paulo Freire and the Maguerez Arc concepts as a theoretical basis for this framework was based on the understanding that such determinants can inspire the practice change processes that are implicit in the implementation of clinical services provided by pharmacists. Together, these theories provided inspiration and subside to develop all steps of the projects that based the proposal and practical experimentation of this framework.

As an example, the first steps of choosing indicators of structure and process, sensitizing managers, gathering the executing team, assessing patient and professional needs, are related to “observation of reality”, which is described by the problematization method with the Maguerez Arc. Only through this path, combined with a continuous process of dialogue with stakeholders involved in the implementation, it was possible to search for solution hypotheses of observed problems and apply them to reality, which occurred during the execution of services. In other words, training pharmacists, resizing the physical structure, and assisting patients.^19^^,^^20^^,^^37^^,^^38^

The intention of the proposed framework is to support previous tools, as well as to show another way of representing the complexity of the process of implementing research. Therefore, we highlight the dialogue and awareness as essential processes that contribute to a comprehensive understanding of the CPS implementation process. As elements that differ ReflACTION framework from other existing proposals in the literature, we mention that this is the only described experience that emerged from practical experiences of healthcare providers and researchers of the Brazilian healthcare system, that was tested in several different services such as drug dispensing, pharmacotherapeutic follow-up, and medication reconciliation, in different levels of healthcare assistance and inspired by the problematization method with the Maguerez Arc. ReflACTION framework emerges from the observation of reality, its challenges and problems, and comes back to reality to implement and assess the effectiveness of solution hypotheses.

Practical lessons of the framework application show that there are many factors that influence the implementation of CPS. These factors may be pointed out by attitudinal, administrative, technical, and political determinants and may be observed as barriers or facilitators. Furthermore, we observed that, although the global efforts to insert pharmacists in the healthcare systems, there are situations where these professionals are not totally included in the organization chart of the healthcare scenarios, which, through observation of reality, evokes the need for elucidation of strategies that decrease the damage related to this situation and insert pharmacists and pharmaceutical services into the multiprofessional healthcare team. According to the literature, this approach would facilitate the integral healthcare, through the rational use of drugs. Finally, based on these experiences, the success of these services can only be achieved by raising awareness among patients and managers and, above all, by applying strategies to train and change pharmacists' attitudes.^21^^,^^35^^,^^37^^,^^38^^,^^53^^,^^54^

Thereby, the ReflACTION framework has the potential to help practitioners understand the process of implementing innovations and move efforts towards the positive transformation of reality. In addition, it has the advantage of starting from reality (practice scenario), so the implementation team may reflect on reality problems of each setting and seek solutions that might positively transform it. Thus, this research is still seeking to increase the understanding of implementing an innovation, especially in CPS implementation. Such investigation may help practitioners to plan and implement effective changes, especially in low- and middle-income countries.

## 5 Conclusion

The ReflACTION framework may be used to describe and guide implementation processes as well as help to understand their outcomes. The proposed framework does not have the intention of developing a gold-standard theoretical model to explain the complexity of the implementation process. In turn, it presents another way of looking through this process, having dialogicity, awareness and problematization as key elements. Our model is based on scientific literature and previous experiences of CPS implementation in different settings of a country with peculiar challenges related to its healthcare system and pharmaceutical profession. We expect the ReflACTION framework continue to evolve as researchers use it and contribute to the knowledge base and ultimately achieve desired patient outcomes. As a future direction, this framework can be useful for implementing CPS, especially in developing countries, in places where the social roles of pharmacists are not yet fully defined, or in the context of public health systems. Therefore, it contributes to the development of the profession and, above all, helps to establish clinical, humanistic, and economic goals among users of medicines and health services.

## Ethics approval and consent to participate

Not applicable.

## Consent for publication

Not applicable.

## Availability of data and materials

The datasets used and/or analyzed during the current study are available from the corresponding author on reasonable request.

## Funding

This study was financed in part by the “Coordenação de Aperfeiçoamento de Pessoal de Nível Superior – Brasil (CAPES) - Finance Code 001”. The funder had no role in the design of the study and collection, analysis, and interpretation of data nor in writing the manuscript.

## CRediT authorship contribution statement

**Kérilin Stancine Santos Rocha:** Writing – review & editing, Writing – original draft, Visualization, Methodology, Investigation, Formal analysis, Data curation, Conceptualization. **Sabrina Cerqueira-Santos:** Writing – review & editing, Writing – original draft, Visualization, Resources, Methodology, Investigation, Formal analysis, Data curation, Conceptualization. **Genival Araújo dos Santos-Júnior:** Writing – review & editing, Writing – original draft, Visualization, Resources, Methodology, Investigation, Data curation, Conceptualization. **Lincoln Marques Cavalcante-Santos:** Writing – review & editing, Writing – original draft, Visualization, Resources, Methodology, Investigation, Formal analysis, Data curation, Conceptualization. **Fernando de Castro Araújo-Neto:** Writing – review & editing, Writing – original draft, Visualization, Resources, Methodology, Investigation, Formal analysis, Data curation, Conceptualization. **Fernanda Oliveira Prado:** Writing – review & editing, Writing – original draft, Visualization, Resources, Methodology, Investigation, Formal analysis, Data curation, Conceptualization. **Giselle de Carvalho Brito:** Writing – review & editing, Writing – original draft, Visualization, Supervision, Resources, Methodology, Investigation, Formal analysis, Data curation, Conceptualization. **Divaldo Pereira de Lyra-Jr.:** Writing – review & editing, Writing – original draft, Visualization, Supervision, Resources, Methodology, Investigation, Formal analysis, Data curation, Conceptualization, Project administration.

## Declaration of competing interest

The authors declare that they have no competing interests.
